# Morphological and phylogenetic analyses reveal a new genus and two new species of Tubakiaceae from China

**DOI:** 10.3897/mycokeys.84.73940

**Published:** 2021-11-22

**Authors:** Zhaoxue Zhang, Taichang Mu, Shubin Liu, Rongyu Liu, Xiuguo Zhang, Jiwen Xia

**Affiliations:** 1 Shandong Provincial Key Laboratory for Biology of Vegetable Diseases and Insect Pests, College of Plant Protection, Shandong Agricultural University, Taian, 271018, China Shandong Agricultural University Tai'an China

**Keywords:** multigene phylogeny, new genus, new species, taxonomy, *Tubakia*

## Abstract

Species of Tubakiaceae have often been reported as plant pathogens or endophytes, commonly isolated from a wide range of plant hosts. The isolated fungi were studied through a complete examination, based on multilocus phylogenies from combined datasets of ITS/LSU/*rpb2* and ITS/*tef1*/*tub2*, in conjunction with morphological characteristics. Five strains isolated from *Lithocarpusfohaiensis* and *Quercuspalustris* in China represented a new genus of Tubakiaceae, *Obovoideisporodochium* and three species, viz. *Obovoideisporodochiumlithocarpi* sp. nov., *Tubakialushanensis* sp. nov. and *T.dryinoides*.

## Introduction

Diaporthales represents an important order in Sordariomycetes containing taxa that are mainly isolated as endophytes, saprobes or plant pathogens on various hosts (Fan et al. 2018). Tubakiaceae is a family in Diaporthales, which has been studied in recent years by Braun et al. (2018) by incorporating morphological and molecular data with appropriate genes to resolve species limitations in the family. Tubakiaceae currently comprises eight genera including *Apiognomonioides* U. Braun et al., *Involutiscutellula* U. Braun & C. Nakash., *Oblongisporothyrium* U. Braun & C. Nakash., *Paratubakia* U. Braun & C. Nakash., *Racheliella* Crous & U. Braun, *Saprothyrium* U. Braun et al., *Sphaerosporithyrium* U. Braun et al. and *Tubakia* B. Sutton (Braun et al. 2018).

*Tubakia*, the type genus of Tubakiaceae, was introduced by Sutton (1973). Species of *Tubakia* are endophytes in leaves and twigs of many tree species, but can also cause conspicuous leaf symptoms as plant pathogens (Harrington et al. 2012; Harrington & McNew 2016, 2018; Braun et al. 2018). The genus is characterised by unique pycnothyria, consisting of pigmented, radiating, seta-like cells (scutellum) on top of a columella, with small phialides on the underside of the scutellum producing ellipsoid, hyaline to brown conidia that are forced out from under the pycnothyrium for rain dispersal (Harrington & McNew 2018). Some species produce a second type of much smaller conidia (microconidia), either in “normal” pycnothyria or in separate, mostly smaller pycnothyria (Braun et al. 2018).

Saccardo (1913) introduced the genus *Actinopelte* for *A.japonica*, a scutellate fungus found in Japan on *Castaneacrenata* (= *C.pubinervis*). Saccardo (1913) confused the large conidia of this species with asci, which was clarified and corrected by Theissen (1913) who provided a detailed discussion, description and illustration (Theissen 1913) of *A.japonica*. Von Höhnel (1925) revisited *Actinopelte*, added a new species, *A.americana* and introduced the new combination *A.dryina*, based on *Leptothyriumdryinum*. Yokoyama & Tubaki (1971) discussed the history of this genus in detail, published results of comprehensive examinations of Japanese collections *in vivo* and *in vitro* and described *A.castanopsidis*, *A.rubra* and *A.subglobosa*, based on Japanese collections. Since Saccardo’s *Actinopelte* turned out to be illegitimate (later homonym of *Actinopelte* Stitzenb. 1861), Sutton (1973) introduced the replacement name *Tubakia* and reallocated all species recognised and treated in Yokoyama & Tubaki (1971) to this genus. Twenty-one additional *Tubakia* species have subsequently been described including fifteen new *Tubakia* species and six combinations in *Tubakia* species (Yun & Rossman 2011; Harrington et al. 2012; Braun et al. 2014; Harrington & McNew 2018; Senanayake et al. 2017; Braun et al. 2018; Yun & Kim 2020).

During field trips to collect plant pathogens causing leaf spots symptoms in China, several specimens associated with typical diaporthalean symptoms were collected from various tree hosts, i.e. *Betuladahurica* (Betulaceae), *Juglansregia* (Juglandaceae), *Prunusdavidiana* (Rosaceae), *Lithocarpusfohaiensis*, *Quercusmongolica* and *Q.palustris* (Fagaceae). Based on morphological analyses as well as phylogenetic data, this study presents a new genus of Tubakiaceae, *Obovoideisporodochium* and three species, viz. *Obovoideisporodochiumlithocarpi* sp. nov., *Tubakialushanensis* sp. nov. and *T.dryinoides* from diseased leaves of *L.fohaiensis* or *Q.palustris*.

## Materials and methods

### Isolation and morphological studies

The samples were collected from the Shandong and Yunnan Provinces, China. The strains were isolated from diseased leaves of *Lithocarpusfohaiensis* and *Quercuspalustris* using tissue isolation methods. Tissue fragments (5 mm × 5 mm) were taken from the margin of leaf lesions and surface-sterilised by consecutively immersing in 75% ethanol solution for 1 min, 5% sodium hypochlorite solution for 30 s and then rinsing in sterile distilled water for 1 min. The pieces were dried with sterilised paper towels and placed on potato dextrose agar (PDA). All the PDA plates were incubated in a biochemical incubator at 25°C for 2–4 days. The colonies from the periphery were picked out and inoculated on to new PDA plates. Colony photos after 7 days and 15 days were taken by a digital camera (Canon Powershot G7X). Micromorphological characters were observed using an Olympus SZX10 stereomicroscope and Olympus BX53 microscope, all fitted with Olympus DP80 high definition colour digital cameras to photo-document fungal structures. All fungal strains were stored in 10% sterilised glycerine at 4°C for further studies. The holotype specimens are deposited in the Herbarium of Plant Pathology, Shandong Agricultural University (HSAUP). Ex-type cultures are deposited in the Shandong Agricultural University Culture Collection (SAUCC). Taxonomic information of the new taxa was submitted to MycoBank (http://www.mycobank.org).

### DNA extraction and amplification

Genomic DNA was extracted from fungal mycelia grown on PDA, using a modified cetyltrimethylammonium bromide (CTAB) protocol as described in Guo et al. (2000). The internal transcribed spacer regions with intervening 5.8S nrRNA gene (ITS), the partial large subunit (LSU) nrRNA gene, part of the beta-tubulin gene region (*tub2*), partial translation elongation factor 1-alpha (*tef1*) and partial RNA polymerase II second largest subunit (*rpb2*) genes were amplified and sequenced by using the primer pairs ITS5/ITS4 (White et al. 1990), LR0R/LR5 (Rehner & Samuels 1994; Vilgalys & Hester 1990), Bt2a/Bt2b (Glass & Donaldson 1995), EF1-728F/EF-2 (O’Donnell et al. 1998; Carbone & Kohn 1999) and f*rpb2*-5F/f*rpb2*-7cR (Liu et al. 1999; Sung et al. 2007).

The PCR was performed using an Eppendorf Master Thermocycler (Hamburg, Germany). Amplification reactions were performed in a 25 μl reaction volume, which contained 12.5 μl Green Taq Mix (Vazyme, Nanjing, China), 1 μl of each forward and reverse primer (10 μM stock) (Biosune, Shanghai, China) and 1 μl template genomic DNA in amplifier, adjusted with distilled deionised water to a total volume of 25 μl. The PCR parameters were as follows: 94°C for 5 min, followed by 35 cycles of denaturation at 94°C for 30 s, annealing at a suitable temperature for 50 s, extension at 72°C for 1 min and a final elongation step at 72°C for 10 min. The annealing temperatures for the genes were 55°C for ITS, 52°C for LSU, 53°C for *tub2*, 48°C for *tef1* and 56°C for *rpb*2. The PCR products were separated with the 1% agarose gel, with added GelRed and UV light used to visualise the fragments. Sequencing was done bi-directionally, conducted by the Biosune Company Limited (Shanghai, China). Consensus sequences were obtained using MEGA v. 7.0 (Kumar et al. 2016). All sequences generated in this study were deposited in GenBank (Table 1).

**Table 1.** Species and GenBank accession numbers of DNA sequences used in this study. New sequences in bold.

**Species**	**Voucher^1^**	**Host/Substrate**	**Country**	**GenBank accession number**				
				**ITS**	**LSU**	***tef1***	***tub2***	***rpb2***
*Greeneriauvicola*	FI12007	‒	Uruguay	HQ586009	GQ870619	‒	‒	‒
*Involutiscutellularubra*	CBS 192.71*	*Quercusphillyraeoides*	Japan	MG591899	MG591993	MG592086	MG592180	MG976476
	MUCC 2303	*Quercusphillyraeoides*	Japan	MG591900	MG591994	MG592087	MG592181	MG976477
	ATCC 22473	*Quercusphillyraeoides*	Japan	MG591901	MG591995	MG592088	‒	MG976478
*Oblongisporothyriumcastanopsidis*	CBS 124732	*Castanopsiscuspidata*	Japan	MG591849	MG591942	MG592037	MG592131	MG976453
	CBS 189.71*	*Castanopsiscuspidata*	Japan	MG591850	MG591943	MG592038	MG592132	MG976454
***Obovoideisporodochiumlithocarpi***	SAUCC 0748*	***Lithocarpusfohaiensis***	**China**	**MW820279**	**MW821346**	**MZ996876**	**MZ962157**	**MZ962155**
	**SAUCC 0745**	***Lithocarpusfohaiensis***	**China**	**MW820280**	**MW821347**	**MZ996877**	**MZ962158**	**MZ962156**
*Paratubakiasubglobosa*	CBS 124733	*Quercusglauca*	Japan	MG591913	MG592008	MG592102	MG592194	MG976489
	CBS 193.71*	*Quercusglauca*	Japan	MG591914	MG592009	MG592103	MG592195	MG976490
*Paratubakiasubglobosoides*	MUCC 2293*	*Quercusglauca*	Japan	MG591915	MG592010	MG592104	MG592196	MG976491
*Racheliellawingfieldiana*	CBS 143669*	*Syzigiumguineense*	Africa	MG591911	MG592006	MG592100	MG592192	MG976487
*Sphaerosporithyriummexicanum*	CPC 32258	*Quercuseduardi*	Mexico	MG591895	MG591989	MG592082	MG592176	‒
	CPC 33021*	*Quercuseduardi*	Mexico	MG591896	MG591990	MG592083	MG592177	MG976473
*Tubakiaamericana*	CBS 129014	*Quercusmacrocarpa*	USA	MG591873	MG591966	MG592058	MG592152	MG976449
*Tubakiacalifornica*	CPC 31505*	*Quercuskelloggii*	USA	MG591835	MG591928	MG592023	MG592117	MG976451
*Tubakiadryina*	CBS 112097*	*Quercusrobur*	Italy	MG591851	MG591944	MG592039	MG592133	MG976455
*Tubakiadryinoides*	**SAUCC 1924**	***Quercuspalustris***	**China**	**MW784842**	**MW784852**	**MW842260**	**MW842263**	**MW842266**
	CBS 329.75	*Quercus* sp.	France	MG591874	MG591967	MG592059	MG592153	MG976458
	MUCC2290	*Castaneacrenata*	Japan	MG591876	MG591968	MG592061	MG592155	MG976459
	MUCC2291	*Castaneacrenata*	Japan	MG591877	MG591969	MG592062	MG592156	MG976460
	MUCC2292*	*Quercusphillyraeoides*	Japan	MG591878	MG591970	MG592063	MG592157	MG976461
*Tubakiahallii*	CBS 129013	*Quercusstellata*	USA	MG591880	MG591972	MG592065	MG592159	MG976462
*Tubakiaiowensis*	CBS 129012*	*Quercusmacrocarpa*	USA	MG591879	MG591971	MG592064	MG592158	‒
*Tubakiajaponica*	ATCC 22472*	*Castaneacrenata*	Japan	MG591886	MG591978	MG592071	MG592165	MG976465
*Tubakiakoreana*	KCTC46072	*Quercusmongolica*	South Korea	KP886837	‒	‒	‒	‒
*Tubakialiquidambaris*	CBS 139744	*Liquidambarstyraciflua*	USA	MG605068	MG605077	MG603578	‒	‒
***Tubakialushanensis***	**SAUCC 1921**	***Quercuspalustris***	**China**	**MW784677**	**MW784850**	**MW842262**	**MW842265**	**MW842268**
	SAUCC 1923*	***Quercuspalustris***	**China**	**MW784678**	**MW784851**	**MW842261**	**MW842264**	**MW842267**
*Tubakiamelnikiana*	CPC 32255*	*Quercuscanbyi*	Mexico	MG591893	MG591987	MG592080	MG592174	MG976472
*Tubakiaoblongispora*	MUCC 2295*	*Quercusserrata*	Japan	MG591897	MG591991	MG592084	MG592178	MG976474
*Tubakiaparadryinoides*	MUCC 2294*	*Quercusacutissima*	Japan	MG591898	MG591992	MG592085	MG592179	MG976475
*Tubakiaseoraksanensis*	CBS 127490*	*Quercusmongolica*	South Korea	MG591907	KP260499	MG592094	MG592186	‒
	CBS 127491	*Quercusmongolica*	South Korea	HM991735	KP260500	MG592095	MG592187	MG976484
*Tubakiasierrafriensis*	CPC 33020	*Quercuseduardi*	Mexico	MG591910	MG592005	MG592099	MG592191	MG976486
*Tubakia* sp.	CBS 115011	*Quercusrobur*	Netherlands	MG591912	MG592007	MG592101	MG592193	MG976488
*Tubakiasuttoniana*	CBS 639.93	*Quercus* sp.	Italy	MG591921	MG592016	MG592110	MG592202	MG976493

^1^ Isolates marked with “*” are ex-type or ex-epitype strains.

## Phylogeny

The generated consensus sequences for each gene were subjected to megablast searches to identify closely-related sequences in the NCBI’s GenBank nucleotide database (Zhang et al. 2000). For the ITS-LSU-*rpb2* and ITS-*tef1*-*tub2* analyses, subsets of sequences from the alignments of Braun et al. (2018) were used as backbones. Newly-generated sequences in this study were aligned with additional related sequences downloaded from GenBank (Table 1) using MAFFT 7 online service with the Auto strategy (Katoh et al. 2019, http://mafft.cbrc.jp/alignment/server/). To establish the identity of the isolates at species level, phylogenetic analyses were conducted, first individually for each locus and then as combined analyses (ITS-LSU-*rpb2* and ITS-*tef1*-*tub2*).

Phylogenetic analyses were based on Maximum Likelihood (ML) and Bayesian Inference (BI) for the multilocus analyses. For BI, the best evolutionary model for each partition was determined using MrModelTest v. 2.3 (Nylander 2004) and incorporated into the analyses. ML and BI were run on the CIPRES Science Gateway portal (https://www.phylo.org/) (Miller et al. 2012) using RAxML-HPC2 on XSEDE v. 8.2.12 (Stamatakis 2014) and MrBayes on XSEDE v. 3.2.7a (Huelsenbeck & Ronquist 2001; Ronquist & Huelsenbeck 2003; Ronquist et al. 2012), respectively. For the ML analyses, the default parameters were used and BI was carried out using the rapid bootstrapping algorithm with the automatic halt option. Bayesian analyses included four parallel runs of 5,000,000 generations, with the stop rule option and a sampling frequency of 50 generations. The burn-in fraction was set to 0.25 and posterior probabilities (PP) were determined from the remaining trees. All resulting trees were plotted using FigTree v. 1.4.4 (http://tree.bio.ed.ac.uk/software/figtree) and the layout of the trees was done in Adobe Illustrator CC 2019.

## Result

### Phylogenetic analyses

#### ITS/LSU/*rpb2 phylogeny*

**Figure 1.**
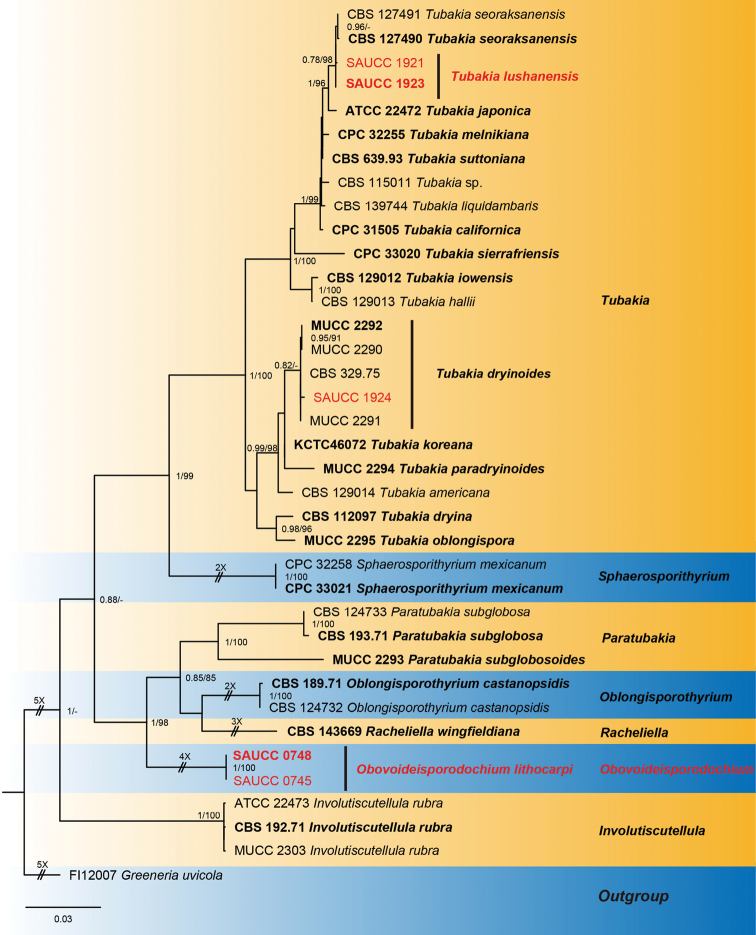
Phylogram of Tubakiaceae, based on the concatenated ITS, LSU and *rpb2* sequence alignment. The BI and ML bootstrap support values above 0.74 and 74% are shown at the first and second position, respectively. The tree is rooted to *Greeneriauvicola* (culture FI12007) and ex-type cultures are indicated in bold face. Strains from the current study are in red. Some branches were shortened for layout purposes – these are indicated by two diagonal lines with the number of times a branch was shortened indicated next to the lines.

The alignment contained 37 isolates representing *Tubakia* and allied taxa and a strain of *Greeneriauvicola* (FI12007) was used as outgroup. The final alignment contained a total of 2459 characters used for the phylogenetic analyses, including alignment gaps, viz. ITS: 1–676, LSU: 677–1545, *rpb2*: 1546–2459. Of these characters, 1858 were constant, 115 were variable and parsimony-uninformative and 486 were parsimony-informative. MrModelTest recommended that the Bayesian analysis should use Dirichlet base frequencies for the ITS, LSU and *rpb2*. The GTR+I+G model was proposed for ITS, LSU and *rpb2*. The MCMC analysis of the three concatenated genes, run for 700,000 generations, resulted in 14,001 trees. The initial 3500 trees, representative of the analysis burn-in phase, were discarded, while the remaining trees were used to calculate posterior probabilities in the majority rule consensus trees (Fig. 1; first value: PP > 0.74 shown). The alignment contained a total of 744 unique site patterns (ITS: 266, LSU: 128, *rpb2*: 350). The topology of the ML tree confirmed the tree topology obtained from the Bayes analyses and, therefore, only the ML tree is presented (Fig. 1). Bayesian posterior probability (> 0.74) and ML bootstrap support values (> 74%) are shown as first and second position above nodes, respectively. The 37 strains were assigned to 25 species clades, based on the three-gene phylogeny (Fig. 1).

#### ITS/*tef1*/*tub2 phylogeny*

**Figure 2.**
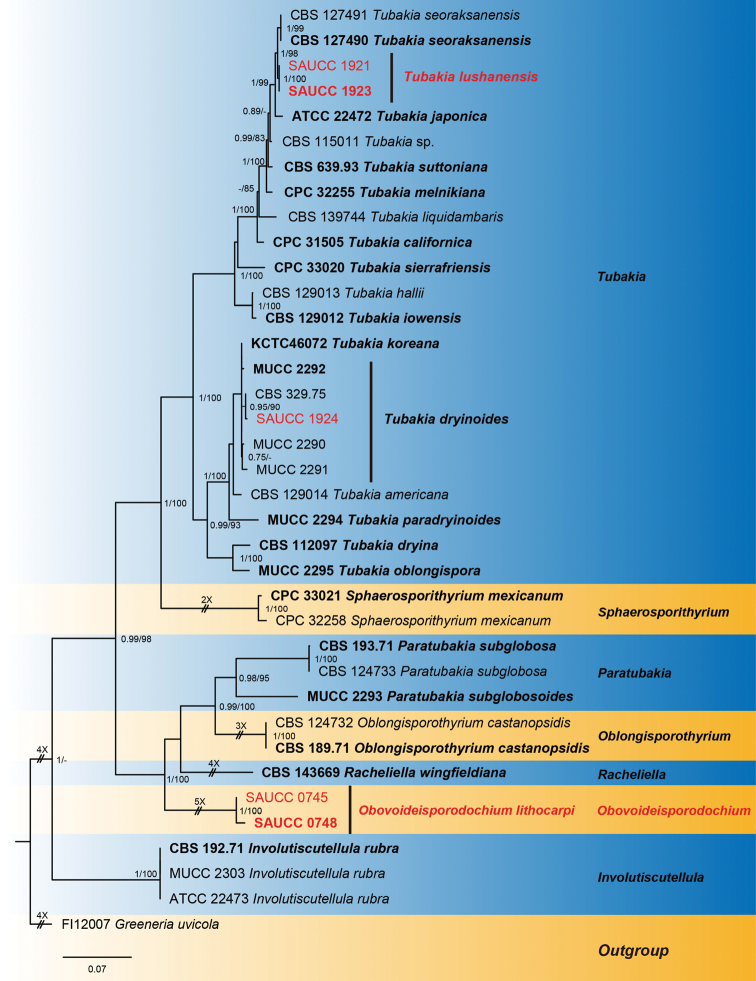
Phylogram of Tubakiaceae, based on the concatenated ITS, *tef1* and *tub2* sequence alignment. The BI and ML bootstrap support values above 0.74 and 74% are shown at the first and second position, respectively. The tree is rooted to *Greeneriauvicola* (culture FI12007) and ex-type cultures are indicated in bold face. Strains from the current study are in red. Some branches were shortened for layout purposes – these are indicated by two diagonal lines with the number of times a branch was shortened indicated next to the lines.

The alignment contained 37 isolates representing *Tubakia* and allied taxa and a strain of *Greeneriauvicola* (FI12007) was used as outgroup. The final alignment contained a total of 1939 characters used for the phylogenetic analyses, including alignment gaps, viz. ITS: 1–676, *tef1*: 677–1358, *tub2*: 1359–1939. Of these characters, 1077 were constant, 136 were variable and parsimony-uninformative and 726 were parsimony-informative. MrModelTest recommended that the Bayesian analysis should use Dirichlet base frequencies for the ITS, *tef1* and *tub2* data partitions. The GTR+I+G model was proposed for ITS and HKY+I+G for *tef1* and *tub2*. The MCMC analysis of the three concatenated genes, run for 170,000 generations resulted in 3401 trees. The initial 850 trees, representative of the analysis burn-in phase, were discarded, while the remaining trees were used to calculate posterior probabilities in the majority rule consensus trees (Fig. 2; first value: PP > 0.74 shown). The alignment contained a total of 997 unique site patterns (ITS: 266, *tef1*: 416, *tub2*: 315). The topology of the ML tree confirmed the tree topology obtained from the Bayes analyses and, therefore, only the ML tree is presented (Fig. 2). Bayesian posterior probability (> 0.74) and ML bootstrap support values (> 74%) are shown as first and second position above nodes, respectively. The 37 strains were assigned to 25 species clades, based on the three-gene phylogeny (Fig. 2).

Based on phylogenetic data (Figs 1 and 2) and morphological analyses, the present study revealed a new genus of Tubakiaceae, *Obovoideisporodochium* and three species, viz. *Obovoideisporodochiumlithocarpi* sp. nov., *Tubakialushanensis* sp. nov. and *T.dryinoides*.

## Taxonomy

### Obovoideisporodochium

Taxon classificationFungiDiaporthalesTubakiaceae

Z. X. Zhang, J. W. Xia & X. G. Zhang gen. nov.

1E1EDA9D-4129-513D-8EAC-E550AB1F4AD1

841103

#### Type species.

*Obovoideisporodochiumlithocarpi* Z. X. Zhang, J. W. Xia & X. G. Zhang

#### Etymology.

Composed of “obovoideisporo-” (obovoid spores) and “-dochium” (referring to the conidioma, i.e. sporodochium).

#### Description.

Genus of Tubakiaceae. Living as endophyte in leaves and causing leaf spots. Asexual morph: mycelium consisting of septate, smooth and hyaline hyphae, thin-walled. Conidiomata sporodochial, appeared within 20 days or longer, formed on agar surface, slimy, pale bluish-green, semi-submerged. Sporodochial conidiophores densely and irregularly branched, bearing apical whorls of 2–3 phialides; sporodochial phialides monophialidic, subulate to subcylindrical, smooth, thin-walled, tapering towards apex, swelling at base. Conidia formed singly, obovoid to ellipsoid, smooth, thin walled, apex obtuse, base with inconspicuous to conspicuous hilum. Sexual morph: unknown.

#### Notes.

In the two phylogenetic trees (Figs 1 and 2), *Obovoideisporodochium* is allied to *Racheliella*, *Oblongisporothyrium* and *Paratubakia*, but forms a separate lineage with full support (PP = 1, ML-BS = 100%), suggesting a genus of its own.

### Obovoideisporodochium lithocarpi

Taxon classificationFungiDiaporthalesTubakiaceae

Z. X. Zhang, J. W. Xia & X. G. Zhang sp. nov.

116C55DA-7F4D-51C3-9577-B3203482C5FB

841104

Fig. 3

#### Type.

China, Yunnan Province: Xishuangbanna Tropical Botanical Garden, Chinese Academy of Sciences, on diseased leaves of *Lithocarpusfohaiensis* (Fagaceae), 11 Sep 2020, Z. X. Zhang, (holotype HSAUP0748, ex-type living culture SAUCC 0748).

**Figure 3.**
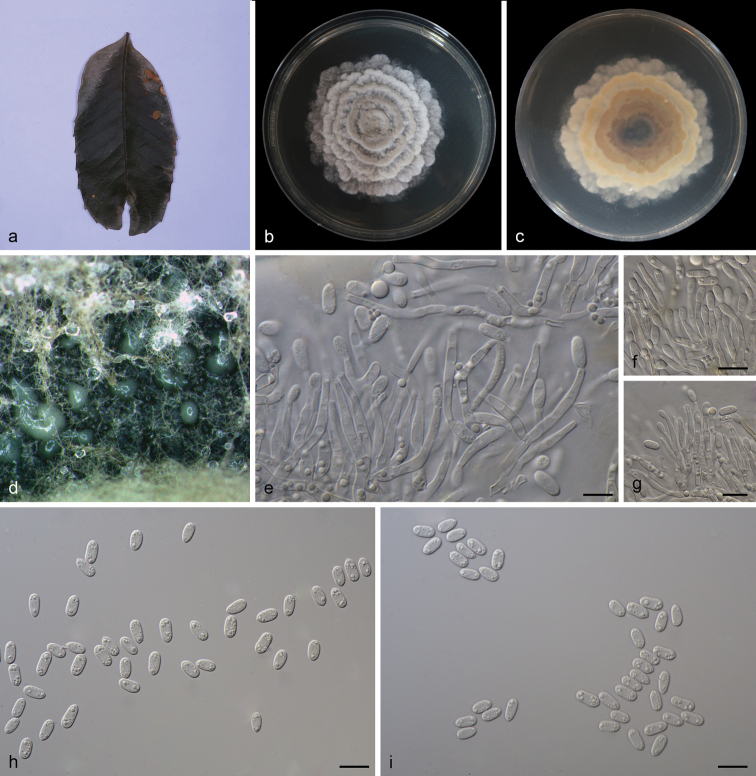
*Obovoideisporodochiumlithocarpi* (SAUCC 0748). **a** infected leaf of *Lithocarpusfohaiensis*; **b** surface of colony after 15 days on MEA; **c** reverse of colony after 15 days on MEA; **d** conidiomata; **e–g** conidiophores, conidiogenous cells and conidia; **h–i** conidia. Scale bars: 10 μm (**e–i**).

#### Etymology.

Name refers to the genus of the host plant *Lithocarpusfohaiensis*.

#### Description.

Asexual morph: mycelium consisting of septate, smooth and hyaline hyphae, thin-walled, 1.0–2.0 μm. Colonies on PDA incubated at 25°C in the dark with an average radial growth rate of 5–6 mm/d and reaching 75–80 mm diam. in 14 d, formed some conspicuous concentric circles, aerial mycelium cottony, white initially, then becoming greyish-sepia. Conidiomata sporodochial, appeared within 20 days or longer, formed on agar surface, slimy, pale bluish-green, semi-submerged. Sporodochial conidiophores densely and irregularly branched, 12.0–26.5 × 1.5–3.0 μm, bearing apical whorls of 2–3 phialides; sporodochial phialides monophialidic, subulate to subcylindrical, 9.5–20.0 × 1.5–3.0 μm, smooth, thin-walled, tapering towards apex, swelling at base. Conidia formed singly, obovoid to ellipsoid, 5.5–8.0 × 2.5–4.0 μm, length/width ratio 1.7–3.1, hyaline, smooth, thin walled, apex obtuse, base with inconspicuous to conspicuous hilum, 0.4–0.9 μm diam. Sexual morph: unknown.

#### Culture characteristics.

Cultures incubated on MEA at 25°C in darkness, attaining 52.0–58.0 mm diam. after 14 d (growth rate 3.5–4.0 mm diam./d), grey-white to creamy white with irregular margin, spread like petals from the inside and outside, reverse dark to light brown, distributed in an irregular circle. Conidial formation not observed.

#### Additional specimen examined.

China, Yunnan Province: Xishuangbanna Tropical Botanical Garden, Chinese Academy of Sciences, on diseased leaves of *Lithocarpusfohaiensis* (Fagaceae), 11 Sep 2020, Z. X. Zhang, HSAUP0745; living culture SAUCC 0745.

#### Notes.

In the two phylogenetic trees (Figs 1 and 2), *Obovoideisporodochiumlithocarpi* is related to *Racheliellawingfieldiana*, *Oblongisporothyriumcastanopsidis*, *Paratubakiasubglobosa* and *P.subglobosoides*, but forms a separate single species lineage with full support (PP = 1, ML-BS = 100%). Furthermore, the conidia of *O.lithocarpi* (5.5–8.0 μm × 2.5–4.0 μm) are smaller than those of *R.wingfieldiana* (11.0–15.0 μm × 6.5–7.5 μm), *Ob.castanopsidis* (14.0–17.0 μm × 7.0–9.5 μm), *P.subglobosa* (10.0–13.0 μm × 8.0–11.0 μm) and *P.subglobosoides* (10.0–12.5 μm × 5.5–10.0 μm) and *Racheliella*, *Oblongisporothyrium* and *Paratubakia* spp. form crustose conidiomata and true pycnothyria.

### Tubakia lushanensis

Taxon classificationFungiDiaporthalesTubakiaceae

Z. X. Zhang, J. W. Xia & X. G. Zhang sp. nov.

32A38215-20E8-5842-8BC2-3BF7497EE959

841105

Fig. 4

#### Type.

China, Shandong Province: Zibo Lushan National Forest Park, on diseased leaves of *Quercuspalustris* Münchh (Fagaceae), 20 Sep 2020, Z. X. Zhang, (holotype HSAUP1923, ex-type living culture SAUCC 1923).

**Figure 4.**
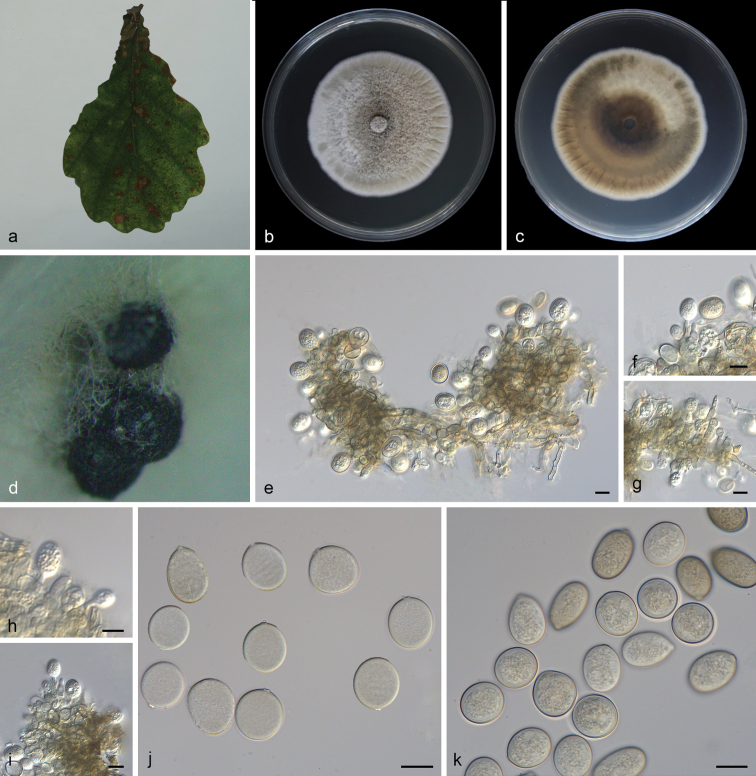
*Tubakialushanensis* (SAUCC 1923). **a** diseased leaf of *Quercuspalustris*; **b** surface of colony after 15 days on MEA; **c** reverse of colony after 15 days on MEA; **d** conidiomata; **e–i** conidiogenous cells with conidia; **j–k** conidia. Scale bars: 10 μm (**e–k**).

#### Etymology

. Named after the type locality, Lushan National Forest Park.

#### Description.

Asexual morph: Leaf spots irregular, occurring on leaf veins and at leaf edges. Colonies on PDA incubated at 25°C in the dark with an average radial growth rate of 5–7 mm/d and occupying an entire 90 mm Petri dish in 14 d, forming some conspicuous concentric circles, aerial mycelium cottony, white initially, then becoming greyish-sepia. Conidiomata pycnidial, usually globose or subglobose when viewed from above, formed on agar surface, black, semi-submerged, up to 200 μm diam. Pycnidial wall composed of an outer layer of yellow-brown, thick-walled textura angularis and an inner layer with hyaline, thin-walled cells. Conidiophores reduced to conidiogenous cells lining the inner cavity, ampulliform or flask-shaped, smooth, hyaline, 9.0–15.0 μm × 2.0–4.0 μm. Conidia solitary, globose to irregular globose, ellipsoid to broad ellipsoid, 10.0–18.0 μm × 7.5–16.0 μm, length/width ratio 1.0–1.7, slightly lighter and wall thin when immature, slightly darker and wall thickened when ripening, smooth, apex rounded, base with peg-like hila, 1.3–2.3 μm diam. Microconidia not observed. Sexual morph not observed.

#### Culture characteristics.

Cultures incubated on MEA at 25°C in darkness, attaining 52.0–56.0 mm diam. after 14 d (growth rate 3.7–4.0 mm diam./d), creamy white to pale brown with regular margin, grey near the centre and hyphae clusters, reverse brown to dark brown rings, heterogeneous colour, with creamy-white edge. Conidial formation not observed.

#### Additional specimen examined.

China, Shandong Province: Zibo Lushan National Forest Park, on diseased leaves of *Quercuspalustris* Münchh. (Fagaceae), 20 Sep 2020, Z. X. Zhang, HSAUP1921; living culture SAUCC 1921.

#### Notes.

The phylogenetic analysis of a combined three-gene alignment (ITS, *tef1* and *tub2*) showed that *T.lushanensis* formed an independent clade and is phylogenetically distinct from its closest sister species *T.seoraksanensis*. This species can be distinguished from *T.seoraksanensis* by 65 different nucleotides in the concatenated alignment (21/628 in the ITS, 31/581 in the *tef1* and 13/521 in the *tub2*). Morphologically, *T.lushanensis* differs from *T.seoraksanensis* in having smaller conidia (10.0–18.0 μm × 7.5–16.0 μm vs. 13.0–25.0 μm × 10.0–15.0 μm) (Yun & Rossman 2011). Furthermore, the MEA’s colony colour of *T.lushanensis* is different from *T.seoraksanensis* (surface: creamy white, pale brown to grey vs. whitish to pale yellow; reverse: creamy white, brown to dark brown vs. olive brown, light olive brown to yellow; Yun & Rossman 2011). Therefore, we describe this fungus as a novel species.

### Tubakia dryinoides

Taxon classificationFungiDiaporthalesTubakiaceae

C. Nakash., Fungal Systematics and Evolution 1: 80 (2018)

02FA01CA-3350-52C2-AC67-C51314144C22

Fig. 5

#### Description.

Asexual morph: Living as endophyte in leaves, forming distinct leaf lesions, shape and size variable, subcircular to angular-irregular, pale brown to brown. Colonies on PDA incubated at 25°C in the dark with an average radial growth rate of 5–7 mm/d and occupying an entire 90 mm Petri dish in 14 d, forming some conspicuous concentric circles, aerial mycelium cottony, white initially, then becoming greyish-sepia. Conidiomata sporodochial, appeared within 14 days or longer, formed on agar surface, slimy, black, semi-submerged. Sporodochial conidiophores densely and irregularly branched, 11.0–24.0 μm × 1.5–5.0 μm, bearing apical whorls of 2–3 phialides; sporodochial phialides monophialidic, subulate to subcylindrical, 9.0–16.0 μm × 1.5–5.0 μm, smooth, thin-walled, apex obtuse to truncate, sometimes forming indistinct periclinal thickenings. Conidia solitary, ellipsoid to obovoid, 6.5–14.0 μm × 4.0–6.0 μm, wall thin, up to 1.0 μm, hyaline to subhyaline, smooth, apex and base broadly rounded, with inconspicuous to conspicuous basal hilum (frill), occasionally somewhat peg-like and truncate when conspicuous. Microconidia not observed. Sexual morph not observed.

**Figure 5.**
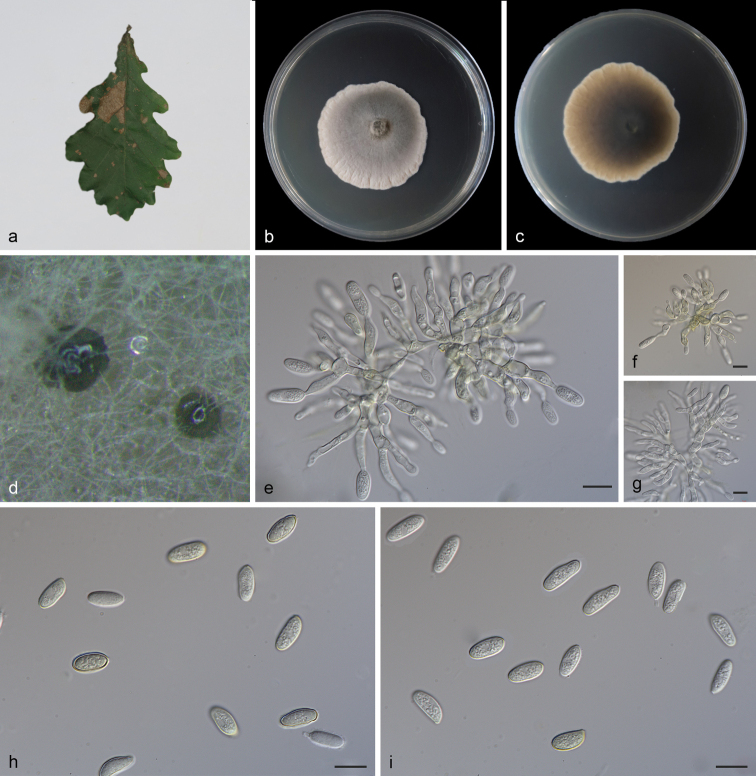
*Tubakiadryinoides* (SAUCC 1924). **a** diseased leaf of *Quercuspalustris*; **b** surface of colony after 15 days on MEA; **c** reverse of colony after 15 days on MEA; **d** conidiomata; **e–g** conidiophores, conidiogenous cells with conidia; **h–i** conidia. Scale bars: 10 μm (**e–i**).

#### Culture characteristics.

Cultures incubated on MEA at 25°C in darkness, attaining 38.0–42.0 mm diam. after 14 d (growth rate 2.7–3.0 mm diam./d), margin scalloped, at first creamy white, grey near the centre, reverse light brown to dark, with olivaceous edge. Conidial formation not observed.

#### Specimen examined.

China, Shandong Province: Zibo Lushan National Forest Park, on diseased leaves of *Quercuspalustris* (Fagaceae), 20 Sep 2020, Z. X. Zhang, HSAUP1924, living culture SAUCC 1924.

#### Notes.

Braun et al. (2018) described *Tubakiadryinoides*, based on morphological and molecular data. The holotype of *T.dryinoides* (NBRC H-11618) was collected from *Quercusphillyraeoides* A. Gray (Braun et al. 2018). In our current research, isolate (SAUCC 1924) collected from diseased leaves of *Quercuspalustris* clustered in the *Tubakiadryinoides* clade by strong support (Figs 1 and 2). We, therefore, consider the isolated strain (SAUCC 1924) as *T.dryinoides*. The conidiomata of *T.dryinoides* is only known from true pycnothyria and the sporodochial conidiomata of the isolated strain (SAUCC 1924) is new for *T.dryinoides* (Braun et al. 2018). Additionally, the conidia of our isolate (SAUCC 1924) is narrower than the original description of *T.dryinoides* (4.0–6.0 μm vs. 5.5–10.0 μm; Braun et al. 2018).

## Discussion

In the study of the phylogenetic affinity and position of *Tubakia* in the Ascomycota hierarchical system, Senanayake et al. (2017) placed this genus in the newly-introduced family Melanconiellaceae. However, the recently-published phylogenetic analyses, including sequence data of the type species of *Tubakia*, confirmed that *Tubakia* warranted a family of its own, Tubakiaceae (Braun et al. 2018) and the description of eight genera including *Apiognomonioides* U. Braun et al., *Involutiscutellula* U. Braun & C. Nakash., *Oblongisporothyrium* U. Braun & C. Nakash., *Paratubakia* U. Braun & C. Nakash., *Racheliella* Crous & U. Braun, *Saprothyrium* U. Braun et al., *Sphaerosporithyrium* U. Braun et al. and *Tubakia* B. Sutton (Braun et al. 2018). The family comprises genera and species with sporodochia, crustose to pustulate pycnidioid stromatic conidiomata and superficial scutellate pycnothyria, monophialidic, colourless, conidiogenous cells, often with collarettes and conidia formed singly, mostly globose to broad ellipsoid-obovoid, aseptate, hyaline to pigmented, often with basal frill or truncate peg-like hilum.

The present study found two new species, one of which represents a novel genus in Tubakiaceae. In order to support the validity of the new species, we followed the guidelines of Braun et al. (2018). Based on ITS/LSU/*rpb2* and ITS/*tef1*/*tub2* molecular data, phylogenetic analyses revealed that two of the obtained isolates (SAUCC 0745 and SAUCC 0748) cluster in a separate lineage, fully supported at genus-level and related to the genera *Racheliella*, *Oblongisporothyrium* and *Paratubakia*. The new genus is named *Obovoideisporodochium* gen. nov. (type species: *Obovoideisporodochiumlithocarpi* sp. nov.). The phylogenetic analyses also revealed that three isolates (SAUCC 1921, SAUCC 1923 and SAUCC 1924) pertain to the genus *Tubakia*. Owing to different nucleotides in the concatenated alignment and morphology, two isolates (SAUCC 1921 and SAUCC 1923) of *Tubakia* were identified as a new species, namely *T.lushanensis* sp. nov, whereas the third isolate (SAUCC 1924) was identified as *T.dryinoides*.

The centre of genetic diversity of *Tubakia* appears to be in East Asia, where *Quercus* and other genera of Fagaceae are the most common hosts (Harrington & McNew 2018). Our study supports this phenomenon well. *Tubakialushanensis* (SAUCC 1921 and SAUCC 1923) and *T.dryinoides* (SAUCC 1924) were isolated from *Quercuspalustris* (Fagaceae), thereby increasing the genetic diversity of *Tubakia* in East Asia.

## Supplementary Material

XML Treatment for Obovoideisporodochium

XML Treatment for Obovoideisporodochium lithocarpi

XML Treatment for Tubakia lushanensis

XML Treatment for Tubakia dryinoides
